# Topics of the Month

**Published:** 1894-07

**Authors:** 


### TOPICS OF THE MONTH.

The New Orleans sewerage system was inaugurated April 18, 1894, with imposing ceremonies in the presence of the mayor, public officials and a large concourse of representative citizens. It seems strange that this delightful and populous city should have surface drainage until the closing years of the nineteenth century. This has been a great drawback to its advancement, but we now predict that within a few years New Orleans will become the most populous and representative city in the South. The establishment of this new system of sewerage is largely due to the efforts of Dr. Joseph Holt, whose fame as a sanitarian is known throughout the world. He has been very properly selected as the president of the new sewerage company, which is a guarantee that its work will be prosecuted with promptitude to a successful issue.

---

This journal has worked hard for many years to deserve the confidence of the profession at home and abroad, and particularly has it aimed to further the interests of medicine in Western New York. Its loyalty to Buffalo and the physicians who reside here cannot be questioned, as its record for nearly fifty years indicates. Our friends will bear testimony to the fact that we rarely refer to ourselves in the Journal pages, but we cannot refrain from printing the delicate and appreciative compliment that the *Kansas City Medical Index* pays us in its issue of May, 1894, in the following paragraph:

> Dr. H. Bronson Gee, of Rochester, N. Y., has begun the publication of the *New York State Medical Reporter*, under the plea that the medical profession of that section of the State has no "medium" in which to present report of the work of excellent local practitioners. One is at a loss to understand why the Buffalo Medical and Surgical Journal (one of the best medical journals in America) should not be regarded as a representative of the physicians of Western New York; but if Dr. Gee can make his journal pay, there is no reason why it should not be published. The initial number is a very creditable one.

---

The Health Department of New York City has issued an important circular relating to tuberculosis. The circular contains the report of Dr. Herman M. Biggs, made last year, together with certain orders and recommendations issued by the health department. The trend of the circular is to furnish information to the people relating to the communicability and preventability of consumption. There is much food for thought in the following paragraph taken from the letter of Dr. Cyrus Edson, chairman of the sanitary committee of board of health, addressed to the Hon. Charles G. Wilson, president, and made a part of the report :

> First. Tuberculosis is a communicable disease and is distinctly preventable. Second. It is acquired by direct transmission of the tubercle bacilli from the sick to the well, usually by means of the dried and pulverized sputum floating as dust in the air. Third. It can be largely prevented by simple and easily applied measures of cleanliness and disinfection.

Among the important features of this report is the action taken by the board of health in regard to apartments occupied by consumptives, which embraces the following order :

> Consumption is a communicable disease. This apartment has been occupied by a consumptive, and has thus become infected. It must not be occupied by persons other than those now residing here until an order of the board of health, directing that it be cleansed and renovated, has been complied with.
>
> Name of occupant ............................................
>
> Floor ..............No. ....................................Street.
>
> This notice must not be removed until the order of the board of health has been complied with.

The department proposes to issue circulars from time to time covering all the various phases of the question of tuberculosis and its prevention for the education of the people in regard to these matters. This is a wise and timely precaution and similar action ought to be taken in Buffalo.

---

The New York Academy of Medicine has appointed a committee, composed of Drs. Daniel Lewis, George Henry Fox and S. T. Armstrong, to confer with the Constitution Convention now in session in regard to such changes in the constitution as may be necessary to protect medical interests in the State of New York. We should like to see this committee augmented by one representing medical societies in Buffalo and Erie county. There are many changes that ought to be made with reference to the conduct of the public health offices throughout the State as well as the office of coroner. It is high time that medical men bestirred themselves on these and other questions pertaining to the public health and the prevention of disease, and we consider it opportune to confer with the constitutional revisors, to the end that any essential changes in the fundamental law may be made under the sanction of that astute body.

---

It would be a great pity to have the annual appropriation for the library of the Surgeon General's office reduced by $3,000, which is proposed by the present appropriation committee of the House of Representatives. It has become pretty well known that the present Congress, feeling itself unable to cope with large questions, has turned to the consideration of petty details, upon which it is wasting much valuable time. But it is beyond the comprehension of even the best friends of this body that it should propose to do so contemptible a thing, as to curtail the valuable educational work of the Surgeon General's office.

---

The second annual report of the Jennie Casseday Infirmary for Women, a private hospital, located at 912 Sixth street, Louisville, Ky., is received. It was established in 1891, and formally opened April 12, 1892, for the treatment of women suffering from diseases peculiar to their sex. Dr. L. S. McMurtry is the surgeon in charge, and during the year covered by this report eighty-nine surgical operations have been made, with but two deaths. A number of these patients were brought to the Infirmary upon matresses, in extreme conditions, but operation was refused in no case, no matter how perilous the state. The two deaths occurred after abdominal section in extremely desperate cases, one suffering with a large cancerous tumor.

This record is one that demonstrates the propriety of special training and preparation for the work, and indicates that a skill has been exercised in this hospital that is fully up to the requirements of the most modern standard of abdominal surgery.

---

The Board of Regents of the University of the State of New York held a meeting at the capitol, in Albany, on Tuesday, June 5, 1894, at which the following-named regents were present: The Chancellor, the Rev. Dr. Anson J. Upson; the Vice-Chancellor, the Rt. Rev. W. C. Doane, Protestant Episcopal Bishop of Albany; Martin I. Townsend, William L. Bostwick, Charles E. Fitch, Orvis H. Warren, Whitlaw Reid, William H. Watson, Hamilton Harris,

Daniel Beach, Willard A. Cobb, Pliny T. Saxton, T. Guilford Smith and the Rev. Father Malone.

An additional interest in the meetings of the regents is taken by physicians since they have become the governing body in relation to State examinations for license to practise medicine. At the June meeting the regents took the following action in regard to the State medical examiners: Drs. William Warren Potter, of Buffalo, W. S. Ely, of Rochester, and Maurice J. Lewi, of New York, were reappointed on the State board. Dr. Asa S. Couch, of Fredonia, was reappointed, and Drs. J. Willis Candee, of Syracuse, and John M. Lee, of Rochester, were appointed on the Homeopathic board. Dr. Lee H. Smith, of Buffalo, was reappointed, and Drs. Orlando Webb Sutton, of Bath, and Melvin H. Nichols, of Wooster, were appointed on the Eclectic board.

We notice that an amendment has been introduced in the constitution convention proposing to merge the Department of Public Instruction with the University, thus doing away with the present two-headed educational system. It would appear from every standpoint that this is a wise measure and ought to receive the unanimous support of the convention. Any plan that removes the administration of educational methods from the whirlpool of politics ought to receive the universal commendation of right-minded citizens.

The Department of Public Instruction should be a bureau in the regents' office, and the superintendent should be appointed by the regents and be made responsible to that body for the faithful performance of his duties.

The work of the regents is everywhere receiving commendation for its high standard in improved educational methods. This is equally true at home and abroad. European educators are constantly watchful of this meritorious work, and are not slow to adopt such portions of it as are applicable.

